# One-out-of-two Quantum Oblivious Transfer based on Nonorthogonal States

**DOI:** 10.1038/s41598-018-32838-9

**Published:** 2018-10-29

**Authors:** Yao-Hsin Chou, Guo-Jyun Zeng, Shu-Yu Kuo

**Affiliations:** Department of Computer Science and Information Engineering, National Chi Nan University, Puli, 54561 Taiwan

## Abstract

This research proposes the first one-out-of-two quantum oblivious transfer (QOT) scheme that does not have a two-level structure and is not subject to Lo’s no-go theorem. Instead, the proposed scheme is a simple and efficient approach based on nonorthogonal states. The nonorthogonality causes one of a pair of messages to be unable to be measured to achieve the irreversible goal of discarding a message, resulting in a one-out-of-two selection effect. The proposed QOT protocol is therefore built directly on quantum resources rather than on a two-level structure in which two classical keys must first be created using quantum resources (all-or-nothing QOT) and then a one-out-of-two protocol is built from there. Furthermore, the proposed protocol allows Alice and Bob to test each other’s loyalty by comparing measurement results. In addition, the relationship with the no-go theorem is discussed in detail; this relationship is often overlooked in other studies. A security analysis demonstrates that the proposed protocol is secure against both external and internal attacks. In addition, an efficiency analysis shows that the proposed protocol is more efficient than other, two-level-structured protocols.

## Introduction

Oblivious transfer (OT) is an important branch of cryptography with many useful and important applications, such as secure computation, bit commitment, remote coin-flipping, and digital contract signing, for which OT protocols are the cryptographic primitives. The two most commonly used OT protocols are the all-or-nothing protocol and the one-out-of-two protocol. All-or-nothing OT was first introduced by Rabin^1^ in 1981. In the all-or-nothing OT protocol, a sender Alice wants to send a secret message, *m* ∈ {0, 1}, to a receiver Bob who has only a 50% probability of receiving *m*. He will either learn the message *m* with 100% reliability or learn nothing about *m*. At the end of all-or-nothing OT, Alice remains oblivious as to whether Bob received the message *m*. Following the proposal of this protocol, Even *et al*.^2^ presented one-out-of-two OT (or it can be abbreviated as 1–2 OT), in which Alice transfers two messages, *m*_0_ and *m*_1_, to Bob, and he can choose only one of them and will have no idea what the other message is. When the one-out-of-two OT protocol is complete, Alice learns nothing about which message Bob selected. In 1988, Crépeau^3^ presented a method for building a one-out-of-two OT protocol by using p-all-or-nothing OT, in which the receiver has a probability *p* of receiving the message *m*, called Crépeau’s reduction. The receiver builds two key sets to represent his choice, *key*_0_ and *key*_1_, one of which he learns with 100% certainty and the other of which he learns with 0% certainty. Based on Bob’s choice *j* ∈ {0, 1}, he asks Alice to encrypt her messages *m*_0_ and *m*_1_ using *key*_*j*_ and $ke{y}_{\bar{j}}$, where $j=0\Rightarrow ke{y}_{0}$ or $j=1\Rightarrow ke{y}_{1}$. Then, Bob can receive *m*_*j*_ under this two-level-structured method.

Classical OT protocols are almost all based on the RSA cryptosystem^4^. However, Shor showed that a quantum algorithm^5^ can be used to break the RSA cryptosystem in polynomial time, which means that such protocols may be unsafe against quantum algorithms. In 1984, Bennett and Brassard proposed the first quantum key distribution protocol^6^, called BB84, thereby initiating the study of quantum cryptography. Researchers later showed that BB84 is unconditionally secure^7–10^ both in theory and in implementation by achieving a one-time pad. The security of quantum cryptography is based on physical laws, unlike that of classical cryptography, which is based on mathematical complexity. This physical basis allows quantum cryptography to easily achieve many goals that were difficult or unthinkable in the past, including unconditional security.

Since the proposal of BB84^6^, researchers have been designing quantum oblivious transfer (QOT) protocols using quantum properties. Crépeau and Kilian^11^ proposed the first all-or-nothing QOT scheme in 1988, and Bennett *et al*.^12^ proposed the first one-out-of-two QOT scheme protected by a quantum error-correcting code in 1992. In 1994, Crépeau^13^ presented a one-out-of-two QOT scheme based on quantum bit commitment (QBC), which guarantees security under the assumption that Bob cannot delay the quantum measurement. In 1995, Yao^14^ further proved that this protocol is secure against coherent measurement if QBC is secure. However, in 1997, Lo^15^ doubted that all one-sided two-party computations (in which two parties must input *i* and *j* to calculate a function *f*(*i*, *j*) but only one of the two parties is allowed to learn the result) may be insecure, including one-out-of-two QOT (the function *f* in one-out-of-two QOT is a selector). This was called Lo’s no-go theorem, and because of the computational equivalent^3,12^ to two OTs, this theorem has caused extreme difficulties in the development of QOT research.

Recent studies have, however, proposed various methods of avoiding Lo’s no-go theorem. In 2002, Shimizu and Imoto^16^ presented an interesting communication method analogous to one-out-of-two QOT with a 50% probability of completing the communication. They^17^ then improved the security of their protocol against entangled pair attacks in 2003. Moreover, in 2006, He and Wang^18^ proposed a secure all-or-nothing QOT scheme using four entangled states, which, as a result, was no longer subject to Lo’s no-go theorem^15^. Consequently, He claimed that Lo’s no-go theorem^15^ did not truly cover all QOT conditions. Thereafter, He^19^ demonstrated that a one-out-of-two QOT scheme built on all-or-nothing QOT protocol using Crépeau’s reduction^3^ also is not subject to Lo’s no-go theorem^15^. The key is that the receiver inputs his choice before the sender inputs her messages *m*_0_ and *m*_1_, causing the functions *f* of the one-out-of-two protocol and Lo’s no-go theorem^15^ to be different.

Following He’s proof^19^, researchers have been designing new one-out-of-two QOT schemes^19^. In 2007, Wei Yang *et al*.^20^ presented a one-out-of-two QOT scheme using tripartite entangled states based on He’s proof^19^ and also showed that this scheme is not covered by the cheating strategy of Lo’s no-go theorem^15^. Li Yang^21^ presented an all-or-nothing QOT scheme using nonorthogonal states, similar to B92^22^, and used it as a basis for constructing a one-out-of-two QOT scheme in 2013. Subsequently, Yu-Guang Yang and his research team, as part of a research effort that began in 2014, have proposed several QOT protocols. They have been testing various schemes for building one-out-of-two QOT protocols using He’s proof^19^. In 2014, they^23^ proposed all-or-nothing and one-out-of-two QOT protocols based on an untrusted third party. In 2015, they^24^ developed an all-or-nothing QOT protocol by analyzing the probability of the qubit state distribution, which led them to propose a method of testing the loyalty of the sender and then to build a one-out-of-two QOT protocol on this basis. They^25^ also designed a one-out-of-two QOT scheme with a two-level structure using BB84^6^ and reduced it to B92^22^ for an all-or-nothing QOT scheme. In addition, they^26^ attempted to use Bell states to achieve the same effect as B92^22^ for one-out-of-two QOT. Furthermore, in 2017, they^27^ proposed a method of using any two nonorthogonal states by cooperatively measuring the qubit sequence and then built a one-out-of-n QOT scheme using this method.

However, these protocols^21,23–27^ all have two-level structures, in which two classical keys are created using an all-or-nothing QOT protocol and then a one-out-of-two QOT protocol is built on top. The two-level structure is clearly inefficient, because many quantum resources are consumed for all-or-nothing QOT instead of being used to transfer the message. In addition, this structure reduces the elasticity and diversity of protocol design because such designs can only follow He’s proof^19^ with minor revisions to the details of the all-or-nothing QOT scheme. In our opinion, He’s proof^19^ not only revealed a different function *f*, which is not subject to Lo’s no-go theorem^15^, based on a two-level structure but also provided a new approach in the sense that if any protocol can achieve the same effect as that of *f* in He’s proof^19^, then it is also covered by He’s proof^19^. In this work, the first one-out-of-two QOT protocol is proposed that is directly based only on the properties of quantum resources, namely, nonorthogonal states, rather than a two-level structure, while also being covered by He’s proof^19^. The key to our protocol is that Bob’s choice is made before Alice inputs her messages *m*_0_ and *m*_1_. The property of nonorthogonality ensures that one of the two messages cannot be measured and thus maintains obliviousness, thereby achieving the same effect as that of *f* in He’s proof^19^. Therefore, our protocol is not only secure (and not subject to Lo’s no-go theorem) but can achieve greater efficiency than protocols^21,23–27^ that are based on a two-level structure.

## Results

This section consists of six subsections, including the preliminaries, the basic idea of our protocol, the proposed protocol itself, its relationship with Lo’s no-go theorem^15^ and He’s proof^19^, and its security and efficiency analyses. The preliminaries introduce the properties of quantum machines and define some notation. Then, the basic idea of the proposed protocol is introduced before the details of the protocol itself, which are described in the subsequent section. Moreover, the relationship among Lo’s no-go theorem^15^, He’s proof^19^ and the proposed protocol is discussed in the subsection titled “Resisting Lo’s cheating strategy^15^”. Finally, security and efficiency analyses are presented in the last two subsections.

### Preliminaries

This subsection introduces the basic definitions of concepts relevant to quantum machines, such as quantum bits, superposition, entanglement, gates, and operations, as well as some properties of quantum machines.

#### Quantum bit

The classical information carrier is called a “bit”. The quantum information carrier is called a “quantum bit”, or a “qubit”. A qubit collapses to certain states of a basis when it is measured. Two bases are commonly used: the Z-basis and the X-basis. The Z-basis is defined as $$|0\rangle =(\begin{array}{c}1\\ 0\end{array})$$ and $$|1\rangle =(\begin{array}{c}0\\ 1\end{array})$$, and the X-basis is defined as $$|\,+\,\rangle =\frac{1}{\sqrt{2}}(\begin{array}{c}1\\ 1\end{array})=\frac{1}{\sqrt{2}}(|0\rangle +|1\rangle )$$ and $$|\,-\,\rangle =\frac{1}{\sqrt{2}}(\begin{array}{c}1\\ -1\end{array})=\frac{1}{\sqrt{2}}(|0\rangle -|1\rangle )$$. A basis is also an orthonormal set.

#### Superposition

Superposition refers to the phenomenon that a qubit can simultaneously exist in both the |0〉 and |1〉 states; i.e., $|\varphi \rangle =\alpha |0\rangle +\beta |1\rangle$, meaning that |*ϕ*〉 will collapse to |0〉 and |1〉 with probabilities of ${\Vert \alpha \Vert }^{2}$ and ${\Vert \beta \Vert }^{2}$, respectively. In addition, the state |−〉 is also considered to be a superposition in the Z-basis. It has a probability of ${\Vert \frac{1}{\sqrt{2}}\Vert }^{2}=\frac{1}{2}$ of collapsing to |0〉 and a probability of ${\Vert -\frac{1}{\sqrt{2}}\Vert }^{2}=\frac{1}{2}$ of collapsing to |1〉.

#### Entanglement

Another important property, entanglement is the phenomenon that qubits cannot exist singly. There are four common entangled states, called Bell states, as shown in Eq. 1. For example, when a state |Φ^+^〉 is measured, as in Eq. 1, the result may be either |00〉_12_ or |11〉_12_, where the subscript indicates the qubit order. As a result, in this case, it is possible to immediately learn the states of two qubits when only one is measured. Einstein referred to this as “spooky action at a distance”.

1 $$\begin{array}{l}{|{{\rm{\Phi }}}^{+}\rangle }_{12}=\frac{1}{\sqrt{2}}{(|00\rangle +|11\rangle )}_{12},\,{|{{\rm{\Phi }}}^{-}\rangle }_{12}=\frac{1}{\sqrt{2}}{(|00\rangle -|11\rangle )}_{12},\\ {|{{\rm{\Psi }}}^{+}\rangle }_{12}=\frac{1}{\sqrt{2}}{(|01\rangle +|10\rangle )}_{12},\,{|{{\rm{\Psi }}}^{-}\rangle }_{12}=\frac{1}{\sqrt{2}}{(|01\rangle -|10\rangle )}_{12}\end{array}$$

#### Quantum gates and operations

Moreover, unitary operations (UU^*^ = U^*^U = *I*) are regarded as gates in quantum computers. There are four common operations, represented by operators called Pauli matrices, which are denoted by {*I*, *X*, *Y*, *Z*}, as shown in Eq. 2. Operations *I* and *Z* cannot be distinguished in the Z-basis, and *Y* and *Z* cannot be distinguished in the X-basis. A single qubit cannot be observed using all four operations, which means that some information is ignored; this is a key element of the proposed protocol. For example, after a *Y* gate, the state |0〉 becomes −|1〉; i.e., a result of |1〉 will be obtained when the qubit is measured. This negative amplitude is called a global phase and cannot be measured. Another important gate is the Hadamard gate, as described in Eq. 2, also called the *H* gate. The *H* gate can be used to convert between two different bases (the Z-basis and the X-basis). For example, after an *H* gate, the state |0〉 (|+〉) *H* becomes |+〉 (|0〉). Table 1 shows the states {|0〉, |1〉, |+〉, |−〉} after the Pauli operations {*I*, *X*, *Y*, *Z*}.

2 $$\begin{array}{lllll}I=(\begin{array}{cc}1 & 0\\ 0 & 1\end{array}), & X=(\begin{array}{cc}0 & 1\\ 1 & 0\end{array}), & Y=(\begin{array}{cc}0 & 1\\ -1 & 0\end{array}), & Z=(\begin{array}{cc}1 & 0\\ 0 & -1\end{array}), & H=\frac{1}{\sqrt{2}}(\begin{array}{cc}1 & 1\\ 1 & -1\end{array})\end{array}$$

**Table 1:** All results after {*I*, *X*, *Y*, *Z*}

States	|0〉	|1〉	|+〉	|−〉
Operations				
*I*	|0〉	|1〉	|+〉	|−〉
*X*	|1〉	|0〉	|+〉	−|−〉
*Y*	−|1〉	|0〉	|−〉	−|+〉
*Z*	|0〉	−|1〉	|−〉	|+〉

### The basic idea

This subsection introduces the basic idea underlying encoding and decoding in the proposed protocol. In this study, the four operations “*I*”, “*X*”, “*Y*” and “*Z*” represent four messages “00”, “10”, “11” and “01”, respectively, for encoding. Each message can be mapped to *m*_0_ and *m*_1_, which represent Alice’s two messages. Because the properties of the two different bases (shown in Table 2) cause a negative amplitude to be unable to be measured (see the last final state in the left-hand part of Table 2), one of the two messages cannot be measured, and which one depends on the basis in which they are prepared (the receiver’s choice). For example, suppose that Bob prepares the state |0〉 and performs either *I* or *H* in accordance with his choice, *j*_0_ or *j*_1_, in order to learn the content of either *m*_0_ or *m*_1_, respectively. In this way, Bob inputs his choice first, and the initial state |0〉 will be either |0〉 or |+〉, depending on his choice. The results are shown in Table 2; after Alice’s operation, if Bob’s choice is *j*_0_ (his initial state is |0〉), he learns *m*_0_ unambiguously (the bold text in the left-hand part of Table 2); otherwise, he learns *m*_1_ (the bold text in the right-hand part of Table 2) unambiguously. As a result, one of the two messages is automatically discarded, thereby achieving the requirements of one-out-of-two QOT.

**Table 2:** The relationship between the qubit states and the encoding.

(m_0_, m_1_)	Alice’s Operation	Bob’s Initial State (j_0_)	Final State	(m_0_, m_1_)	Alice’s Operation	Bob’s Initial State (j_1_)	Final State
(**0**, 0)	*I*	|0〉	|0〉	(0, **0**)	*I*	|+〉	|+〉
(**0**, 1)	*Z*		|0〉	(1, **0**)	*X*		|+〉
(**1**, 0)	*X*		|1〉	(1,** 1**)	*Y*		|−〉
(**1**, 1)	*Y*		**−**|1〉	(0, **1**)	*Z*		|−〉

### The proposed protocol

As seen from the basic idea presented above, the operations *I* and *H* can be regarded as representing Bob’s intentions regarding his choice; this makes the proposed protocol similar to B92^22^, which has been proven unconditionally secure both in theory and in implementation^28,29^, meaning that no one can perfectly identify all states of the qubits without any information from their creator. Another key property is that some operations cannot be distinguished in some bases, which means that it is not possible to identify all operations from a single qubit. The proposed protocol allows Alice and Bob to test each other’s loyalty, because they can check whether the initial and final states are correct. In other words, if they want to lie to each other, it will create errors, which can be discovered when they test each other. Let us give a simple example at the end of every step of the protocol without channel checking. The proposed protocol consists of 7 steps as follows:

**Step 1**. Bob creates a qubit sequence in accordance with his choice intentions *j*_0_ and *j*_1_, which correspond to the states |0〉 and |+〉, respectively. The necessary *I* and *H* gates can be considered as equivalent to his choice intentions in this stage. This sequence must be longer than the OT sequence, which contains all received message qubits as well as qubits for channel checking and for testing Bob’s loyalty. In addition, the channel checking and loyalty testing states are different; the former, also called decoy qubits, belong to {|0〉, |1〉, |+〉, |−〉}, and the latter belong to {|0〉, |+〉}. If *N* denotes the minimum length (at which Bob will receive *N* messages), *M* is the number of channel checking qubits, and *K* is the number of loyalty testing qubits, then the total length of the QOT sequence is *N* + *M* + 2*K*. Bob randomly prepares *M* qubits from {|0〉, |1〉, |+〉, |−〉} (each qubit is independent) and inserts them into his sequence. Subsequently, he also inserts his *N* and 2*K* candidate choice intentions ({|0〉, |+〉}) into his sequence and then sends the sequence to Alice. Let us give a simple example to describe the proposed one-out-of-two QOT protocol without channel checking, suppose that Bob prepares two qubits in |0+〉_12_ (*N*) to represent his choices and an additional two qubits in |0+〉_34_ (2*K*) for loyalty testing. He then sends these four qubits to Alice.

**Step 2**. Once she receives the sequence from Bob, Alice first checks the channel for an eavesdropper (Eve) and then tests Bob’s loyalty. First, she asks Bob to publish the bases and states that he has created. If the error rate is higher than a given threshold, then an Eve is present on the channel, and Alice and Bob abort their communication; otherwise, Alice goes on to test Bob’s loyalty. She discards the qubits for channel checking and then randomly selects several positions and requests that Bob publish his bases. If different results, i.e., ∉ {|0〉, |+〉}, are measured and the error rate is higher than the given error rate, then Bob is considered dishonest, and she aborts this communication; otherwise, she proceeds to the next step. Following the above example, once Alice receives the ordered sequence |0+0+〉_1234_, she randomly chooses a qubit for loyalty testing. Suppose that Alice’s random choice is qubit 4; then, she asks Bob to publish the basis of qubit 4, measures it, and compares the published and measured results. If the error rate is higher than the threshold, then Bob is considered as dishonest; after that, qubit 4 is discarded.

**Step 3**. Since the loyalty test may disturb the order of Bob’s choice intentions, Bob must ask Alice to reorder the qubits. It is for this purpose that 2*K* additional qubits are initially provided to prevent vacancies in the list of choice intentions. In this step, the sequence after reordering represents Bob’s real choices. Following the above example, after the loyalty test, Bob asks Alice to reorder the remaining qubits in the order 21, and the states become |+0〉_21_, with qubit 3 discarded. The resulting state |+0〉_21_ represents Bob’s choices, *j*_1_ and *j*_0_, respectively.

**Step 4**. Alice now inputs her secret messages *m*_0_ and *m*_1_ through the *I*, *X*, *Y* and *Z* operations, corresponding to the combinations “00”, “10”, “11” and “01”, respectively. Following the above example, Alice performs *Z* and *X* in accordance with her messages “01_12_” and “10_34_”, where the subscripts indicate Alice’s classical bit order, on qubits 2 and 1, respectively. This converts the state |+0〉_21_ into |−1〉_21_.

**Step 5**. Alice then randomly inserts decoy qubits from {|0〉, |1〉, |+〉, |−〉} into the sequence for channel checking and sends the sequence to Bob.

**Step 6**. When Bob receives the sequence from Alice, he asks Alice to publish the positions and states of the decoy qubits. If the error rate is higher than the channel error rate, they abort this communication and return to step 1. Otherwise, Bob learns the contents of the classical messages by measuring the qubits with the bases he prepared. Following the above example, Bob performs X- and Z-basis measurements to learn the second and first classical messages, “1” and “1” (01_12_ and 10_34_, where the subscripts indicate Alice’s classical bit order), respectively.

**Step 7**. At the end of this protocol, where Bob has to test Alice’s loyalty to prevent Alice from cheating, she can learn Bob’s choice with the probability 25%, or 29.3% by POVM. Bob chooses some random positions and asks Alice to publish her operations. Bob performs the operations according to Alice’s announcement, in order to recover those qubits into {|0〉, |+〉}. If the error rate of loyalty testing is higher than the threshold, then Alice is considered as dishonest. Following the above example, Bob asks Alice to publish operation (*X*), which performs on qubit 1; then, Bob performs *X* on qubit 1 to recover the state into |0〉.

### Resisting Lo’s cheating strategy

Lo’s no-go theorem^15^ provides a cheating strategy for learning all messages in a one-sided two-party secure computation, which doubts that one-out-of-two QOT is insecure. The key to Lo’s cheating strategy^15^ is requirement (A-i), namely, that “Bob learns *f*(*i*(*m*_0_, *m*_1_), *j*) unambiguously” (the *i*(*m*_0_, *m*_1_) represents a pair of messages in a one-out-of-two QOT scenario), which leads to a probability of 100% that the selected state will collapse. In addition, the result is obtained after reversible operations. As a result, choices can be made repeatedly to learn all messages, as shown in Eq. 3, where ${U}_{{j}_{0}}$, ${U}_{{j}_{1}}$, *G* and |*ϕ*〉 represent two different selected operations, any unitary operation for inputting two messages and any quantum state, respectively. Therefore, once Bob learns the content of a message, he can recover the state *G* × |*ϕ*〉, which is the state after Alice’s input, by applying the selected operation ${U}_{{j}_{k}}$ and its inverse operation ${U}_{{j}_{k}}^{\ast }$, where *k* ∈ {|0〉, |1〉}. In this way, Bob can change his choice and learn the contents of all messages by repeating the above process.

3 $${U}_{{j}_{0}}^{\ast }\cdot {U}_{{j}_{0}}\cdot G\cdot |\varphi \rangle ={U}_{{j}_{1}}^{\ast }\cdot {U}_{{j}_{1}}\cdot G\cdot |\varphi \rangle $$

Definition B corresponds to the one-out-of-two QOT scenario covered by Lo’s proof^15^. Obviously, definition B is a special case of definition A. Definition B describes the case in which Alice inputs her messages first and then Bob inputs his choices. The important point here is that if Bob inputs his choices first and Alice subsequently input her messages, as in definition C (the proposed protocol), this scenario is not equivalent to the function considered in Lo’s proof^15^. This is because the function becomes *f*(*i*(*m*_0_, *m*_1_, *j*), *j*) when Bob inputs his choices first, and *f*(*i*(*m*_0_, *m*_1_, *j*_0_), *j*_1_) is meaningless with respect to *f*(*i*(*m*_0_, *m*_1_, *j*), *j*). Therefore, Bob cannot change *i* from *i*(*m*_0_, *m*_1_, *j*_0_) to *i*(*m*_0_, *m*_1_, *j*_1_) without Alice’s help. Following from the above relation, in the proposed protocol (definition C), the result after Bob’s and Alice’s actions can be expressed as

4 $${U}_{{j}_{0}}^{\ast }\cdot G\cdot {U}_{{j}_{0}}\cdot |0\rangle \ne {U}_{{j}_{1}}^{\ast }\cdot G\cdot {U}_{{j}_{1}}\cdot |0\rangle $$

Here, ${U}_{{j}_{0}}=I$, ${U}_{{j}_{1}}=H$, and *G* ∈ {*I*, *X*, *Y*, *Z*}. Eq. 4 shows that Bob cannot invert the qubit state without possessing information about *G*. Therefore, Bob cannot perform Lo’s cheating strategy^15^. As a result, Bob cannot reverse the effects of his inputs without Alice’s help. The condition of Equation 4 shows that the proposed function *f*(*i*(*m*_0_, *m*_1_, *j*), *j*) is similar to that of He’s proof^19,30^. This proof shows that the order of input of the choices and messages may change the function *f*, which means that this protocol is not subject to Lo’s no-go theorem. In addition, He^30^ has extended the concept of his proof^19^ to the general case; if Alice and Bob interact with each other and Bob cannot eliminate the effects of his operations independently, then the interaction is covered by He’s proof^19^ and resists Lo’s cheating strategy^15^.

- **Definition A: ideal one-sided two-party secure computation**

- **(A-i)** Bob learns *f*(*i*, *j*) unambiguously.
- **(A-ii)** Alice learns nothing about *j* and *f*(*i*, *j*).
- **(A-iii)** Bob learns nothing about *i* more than what logically follows from the values of *j* and *f*(*i*, *j*).

- **Definition B: one-out-of-two OT (Lo’s no-go theorem** ^15^ **)**

- **(B-i)** Alice inputs *i*, which is a pair of messages (*m*_0_, *m*_1_).
- **(B-ii)** Bob inputs *j* = 0 or 1.
- **(B-iii)** At the end of the protocol, Bob learns the content of message *m*_*j*_ but not of the other message ${m}_{\bar{j}}$; i.e., the protocol is an ideal one-sided two-party secure computation, with *f*(*m*_0_, *m*_1_, *j* = 0) = *m*_0_ and *f*(*m*_0_, *m*_1_, *j* = 1) = *m*_1_.
- **(B-iv)** Alice does not know which *m*_*j*_ Bob received.

- **Definition C: the proposed protocol**

- **(C-i)** Bob inputs *j* = 0 or 1 to change the qubit state to {|0〉, |+〉} (Z- or X-basis) in accordance with his choice intention.
- **(C-ii)** Alice inputs her messages *m*_0_ and *m*_1_ using {*I*, *X*, *Y*, *Z*}.
- **(C-iii)** Bob learns the content of either *m*_0_ or *m*_1_ using the basis (Z- or X-basis) he prepared.

## Security Analysis

Two security conditions are considered in this study: security against external and internal attacks. External attacks involve an eavesdropper, Eve, attempting to steal messages without being detected. Internal attacks involve either Alice or Bob attempting to steal the other’s secret information; i.e., Alice wants to learn Bob’s choices, or Bob wants to learn the contents of both of Alice’s messages.

### External Attack

Alice and Bob must ensure that the communication channel between them is secure, because without channel checking or reduced frequency^31^, Eve will be able to illicitly eavesdrop on their messages. In the proposed protocol, several single qubits ∈ {|0〉, |1〉, |+〉, |−〉} are randomly inserted into the transmitted sequence as decoy qubits for channel checking, as described in steps 1 and 5 of the protocol. The positions and states of these qubits are then published and measured to check whether an Eve is present. If the measured results obtained with the same bases are different and the error rate is higher than the channel error rate, then an Eve is present. Two common external attack strategies are the intercept-and-resend attack and the entangling attack. They are discussed below.

#### Intercept-and-resend attack

Eve intercepts all qubits during transmission when the sender sends the qubit sequence to the receiver, measures them to obtain the message contents, and then resends those qubits to the receiver. This action should disturb the states of the qubits, including the decoy qubits, because Eve does not know which bases have been prepared by Alice and Bob. According to the detection rate of BB84^6^, each qubit has a probability of $\frac{1}{4}$ of detecting Eve’s presence, and the detection rate increases with an increasing number of decoy qubits *M*. As a result, the security level can be assessed based on the detection rate by legal agents, *ξ*_1_, as expressed in Eq. 5.

5 $${\xi }_{1}=1-{(\frac{3}{4})}^{M}$$

#### Entangling attack

Eve may instead use a different method that does not disturb the qubit states, namely, the entangling attack. In this attack, she intercepts the transmitted sequence, prepares an ancillary qubit |*E*〉, and performs a unitary operation *U*_*e*_ on the intercepted qubit to entangle it with her qubit |*E*〉 during transmission. The unitary operation *U*_*e*_ is defined as shown in Eq. 6, where |*e*_00_〉, |*e*_01_〉, |*e*_10_〉, and |*e*_11_〉 are four states determined by the unitary operation *U*_*e*_, ${\Vert a\Vert }^{2}+{\Vert b\Vert }^{2}=1$, and ${\Vert c\Vert }^{2}+{\Vert d\Vert }^{2}=1$. If Eve wishes to avoid detection, the operation *U*_*e*_ must satisfy *a* = *d* = 1, *b* = *c* = 0, and |*e*_00_〉 = |*e*_11_〉, and as a result, the proposed protocol ensures that no information can be obtained in this way.

6 $$\begin{array}{l}{U}_{e}(|0\rangle |E\rangle )=a|0\rangle |{e}_{00}\rangle +b|1\rangle |{e}_{01}\rangle \\ {U}_{e}(|1\rangle |E\rangle )=c|0\rangle |{e}_{10}\rangle +d|1\rangle |{e}_{11}\rangle \\ {U}_{e}(|+\rangle |E\rangle )=\frac{1}{\sqrt{2}}(a|0\rangle |{e}_{00}\rangle +b|1\rangle |{e}_{01}\rangle +c|0\rangle |{e}_{10}\rangle +d|1\rangle |{e}_{11}\rangle )\\ =\frac{1}{2}|+\rangle (a|{e}_{00}\rangle +b|{e}_{01}\rangle +c|{e}_{10}\rangle +d|{e}_{11}\rangle )+\frac{1}{2}|-\rangle (a|{e}_{00}\rangle -b|{e}_{01}\rangle +c|{e}_{10}\rangle -d|{e}_{11}\rangle )\\ {U}_{e}(|-\rangle |E\rangle )=\frac{1}{\sqrt{2}}(a|0\rangle |{e}_{00}\rangle +b|1\rangle |{e}_{01}\rangle -c|0\rangle |{e}_{10}\rangle -d|1\rangle |{e}_{11}\rangle )\\ =\frac{1}{2}|+\rangle (a|{e}_{00}\rangle +b|{e}_{01}\rangle -c|{e}_{10}\rangle -d|{e}_{11}\rangle )+\frac{1}{2}|-\rangle (a|{e}_{00}\rangle -b|{e}_{01}\rangle -c|{e}_{10}\rangle +d|{e}_{11}\rangle )\end{array}$$

### Internal Attack

Internal attacks involve the legal agents Alice and Bob attempting to steal each other’s secret information; i.e., Alice wants to learn Bob’s choices, or Bob wants to learn the contents of all messages sent by Alice. Therefore, two conditions must be discussed, namely, Alice’s and Bob’s cheating strategies.

#### Alice’s cheating strategy

There are two conditions to be discussed. The first condition is that Alice has no ability of entanglement. In this condition, Alice only has the ability to perform a single qubit gate such as {*I*, *X*, *Y*, *Z*, *H*} etc., and she has 25% or 29.3% chance to learn Bob’s choices; however, this kind of attack can be always detected in our protocol. The second condition is that Alice has the ability of entanglement. In this condition, Alice has the ability to perform two or more qubit gates, which leads to diverse attacks. However, Bob is also required to have the ability of entanglement to resist attacks from Alice, and a dishonest Alice will be detected by the discussion below.

#### Alice has no ability of entanglement

A dishonest Alice can learn 25% of Bob’s choices, as in B92^22^, because a measurement in the incorrect basis can yield incorrect measurement results that nevertheless help Alice to determine Bob’s initial state. For example, if Bob sends the state 0 to Alice, she has a probability of $\frac{1}{2}$ of using the incorrect basis (X-basis), and when she does so, the incorrect state (|−〉) will be obtained with a probability of $\frac{1}{2}$, resulting in a total probability of $\frac{1}{4}=0.25$ $(\frac{1}{2}\times \frac{1}{2})$. In fact, there is a 29.3% chance that Alice will learn Bob’s choices with POVM {*E*_1_, *E*_2_, *I* − *E*_1_ − *E*_2_} on Bob’s qubit, where

7 $${E}_{1}\equiv \frac{\sqrt{2}}{1+\sqrt{2}}|1\rangle \langle 1|\,{\rm{and}}\,{E}_{2}\equiv \frac{\sqrt{2}}{1+\sqrt{2}}|-\rangle \langle -|.$$

Then, she can unambiguously distinguish states {|0〉, |+〉} with probability

8 $$\langle 0|{E}_{1}|0\rangle =\langle \,+\,|{E}_{2}|\,+\,\rangle =\frac{1}{\sqrt{2}\mathrm{(1}+\sqrt{2})}\approx \mathrm{29.3 \% .}$$

In other word, the remaining 70.7% of Bob’s choices will be unknown, which means that Alice should randomly create several state ∈ {|0〉, |1〉, |+〉, |−〉} to send to Bob. However, she cannot know Bob’s final measurement results, as he does not publish any information about the bases. In other words, for each bit, he will be unable to correctly decrypt with a probability of $\mathrm{(1}-\mathrm{29.3 \% )}\times \frac{1}{2}\times \frac{1}{2}=\mathrm{17.675 \% }$ at the Alice’s loyalty testing stage, which will make him aware of Alice’s dishonesty with

9 $${\xi }_{2}=1-\,{\mathrm{(1}-\mathrm{0.17675)}}^{D},$$

where *ξ*_2_ can be decided by the user through the number of qubit *D* for loyalty testing. Therefore, Bob can detect that Alice is cheating. If Alice does not use POVM, the total detection rate is $\mathrm{(1}-\mathrm{0.25)}\times \frac{1}{2}\times \frac{1}{2}=\mathrm{18.75 \% }$ with a single qubit. Indeed, while the detection rate dropped by 18.75% − 17.675% = 1.075% with POVM, it does not change the number of particles too much.

For a simple example regarding the detection rate with a remaining qubit, which Alice randomly prepared, Alice prepares a qubit in state |0〉 and guesses Bob’s choice. In this case, she can only publish operation {*I*, *Z*} to escape this testing, and there are two branches: **1**. Bob uses Z-basis as his choice; in this case, Alice can always escape the testing; **2**. Bob uses X-basis as his choice; in this case, Bob has a 50% to get |+〉 or |−〉. When he gets |+〉, the operation *Z* cannot restore the state |+〉 to |+〉. Otherwise, when he gets |−〉, operation *I* cannot restore the state |−〉 to |+〉. Therefore, Bob always has a probability to detect Alice’s dishonesty.

#### Alice has the ability of entanglement

A dishonest Alice can prepare Bell states in |Φ^+^〉_*AB*_ to perform a teleportation attack. In this way, she can pass Alice’s loyalty testing, and then, learn Bob’s choices with 25% or 29.3% chance without being detected. For a simple example to explain the teleportation attack, in step 3, the qubit from Bob after Bob’s loyalty testing is called |*φ*〉_*C*_. In step 4, instead of inputting her secret message into qubit *C*, Alice creates a Bell state in ${|{{\rm{\Phi }}}^{+}\rangle }_{AB}$, distributes qubit *B* to Bob to replace qubit *C*, and holds qubit *A*. After that, Alice performs a Bell measurement (a controlled-not gate and a Hadamard gate, which can transfer four Bell states |Φ^+^〉, |Φ^−^〉, |Ψ^+^〉 and |Ψ^−^〉 into |00〉, |10〉, |01〉 and |11〉, respectively), BM for short, on qubit *C* and *A*, and publishes one of four operations *I*, *X*, *Y* or *Z* as her secret message according to BM results |00〉_*CA*_, |01〉_*CA*_, |11〉_*CA*_ and |10〉_*CA*_, respectively. Bob can then perform one of four operations *I*, *Z*, *X* or *Y* to recover state |0〉_*B*_ or |+〉_*B*_ according to a result that Alice published. As shown in Eqs 10 and 11, Bob can always recover the qubit state |0〉_*B*_ or |+〉_*B*_, because the BM results |00〉_*CA*_, |10〉_*CA*_, |01〉_*CA*_ and |11〉_*CA*_ can always match operations *I*, *Z*, *X* and *Y*, respectively.

10 $$\begin{array}{lll}{|0\rangle }_{C}\otimes \frac{1}{\sqrt{2}}{(|00\rangle +|11\rangle )}_{AB} & \mathop{\Rightarrow }\limits^{B{M}_{CA}} & \frac{1}{2}({|00\rangle }_{CA}\otimes {|0\rangle }_{B}+{|10\rangle }_{CA}\otimes {|0\rangle }_{B}\\  &  & +\,{|01\rangle }_{CA}\otimes {|1\rangle }_{B}+{|11\rangle }_{CA}\otimes {|1\rangle }_{B})\end{array}$$

11 $$\begin{array}{lll}{|+\rangle }_{C}\otimes \frac{1}{\sqrt{2}}(|00\rangle +{|11\rangle }_{AB}) & \mathop{\Rightarrow }\limits^{B{M}_{CA}} & \frac{1}{2}({|00\rangle }_{CA}\otimes {|+\rangle }_{B}+{|10\rangle }_{CA}\otimes {|-\rangle }_{B}\\  &  & +\,{|01\rangle }_{CA}\otimes {|+\rangle }_{B}\,-\,{|11\rangle }_{CA}\otimes {|-\rangle }_{B})\end{array}$$

However, without loss of generality, Bob should also have the same entanglement ability as Alice. He can prepare an entangled state in $\frac{1}{\sqrt{2}}{(|00\rangle +|+1\rangle )}_{{A}_{1}{B}_{1}}$ and sends qubit *A*_1_ to Alice. Under normal conditions, we can know that Alice will do honest behavior. After Alice performs an operation in {*I*, *X*, *Y*, *Z*} on qubit *A*_1_, the entangled state will be Eq. 12. We can divide those states into two bases (Eqs 13 and 14), called IY basis and XZ basis, respectively, of which the IY/XZ basis can perfectly distinguish the states after ${I}_{{A}_{1}}$/${X}_{{A}_{1}}$ and ${Y}_{{A}_{1}}$/${Z}_{{A}_{1}}$. Obviously, these two bases are not orthogonal. That is to say, Bob will measure qubits *A*_1_ and *B*_1_ with one of two bases {*IY*, *XZ*} according to Alice’s operations to check Alice’s loyalty.

12 $$\frac{1}{\sqrt{2}}{(|00\rangle +|+1\rangle )}_{{A}_{1}{B}_{1}}\Rightarrow \{\begin{array}{l}\mathop{\to }\limits^{{I}_{{A}_{1}}}\frac{1}{\sqrt{2}}{(|00\rangle +|+1\rangle )}_{{A}_{1}{B}_{1}}=\frac{1}{\sqrt{2}}{(|00\rangle +\frac{1}{\sqrt{2}}|01\rangle +\frac{1}{\sqrt{2}}|11\rangle )}_{{A}_{1}{B}_{1}}\\ \mathop{\to }\limits^{{Y}_{{A}_{1}}}\frac{1}{\sqrt{2}}{(|-1\rangle -|10\rangle )}_{{A}_{1}{B}_{1}}=\frac{1}{\sqrt{2}}{(-|10\rangle +\frac{1}{\sqrt{2}}|01\rangle -\frac{1}{\sqrt{2}}|11\rangle )}_{{A}_{1}{B}_{1}}\\ \mathop{\to }\limits^{{X}_{{A}_{1}}}\frac{1}{\sqrt{2}}{(|10\rangle +|+1\rangle )}_{{A}_{1}{B}_{1}}=\frac{1}{\sqrt{2}}{(|10\rangle +\frac{1}{\sqrt{2}}|01\rangle +\frac{1}{\sqrt{2}}|11\rangle )}_{{A}_{1}{B}_{1}}\\ \mathop{\to }\limits^{{Z}_{{A}_{1}}}\frac{1}{\sqrt{2}}{(|00\rangle +|-1\rangle )}_{{A}_{1}{B}_{1}}=\frac{1}{\sqrt{2}}{(|00\rangle +\frac{1}{\sqrt{2}}|01\rangle -\frac{1}{\sqrt{2}}|11\rangle )}_{{A}_{1}{B}_{1}}\end{array}$$

13 $$\,{\rm{I}}{\rm{Y}}\,{\rm{b}}{\rm{a}}{\rm{s}}{\rm{i}}{\rm{s}}=\{\begin{array}{cccc}(\begin{array}{c}1/\sqrt{2}\\ 1/2\\ 0\\ 1/2\end{array}), & (\begin{array}{c}0\\ 1/2\\ -1/\sqrt{2}\\ -1/2\end{array}), & (\begin{array}{c}1/2\\ 0\\ 1/2\\ -1/\sqrt{2}\end{array}), & (\begin{array}{c}-1/2\\ 1/\sqrt{2}\\ 1/2\\ 0\end{array})\end{array}\}$$

14 $${\rm{X}}{\rm{Z}}\,{\rm{b}}{\rm{a}}{\rm{s}}{\rm{i}}{\rm{s}}=\{\begin{array}{cccc}(\begin{array}{c}0\\ 1/2\\ 1/\sqrt{2}\\ 1/2\end{array}), & (\begin{array}{c}1/\sqrt{2}\\ 1/2\\ 0\\ -1/2\end{array}), & (\begin{array}{c}1/2\\ -1/\sqrt{2}\\ 1/2\\ 0\end{array}), & (\begin{array}{c}1/2\\ 0\\ -1/2\\ 1/\sqrt{2}\end{array})\end{array}\}$$

However, Alice may be dishonest and perform a teleportation attack. Alice creates an entangled state in ${|{{\rm{\Phi }}}^{+}\rangle }_{AB}$ and sends qubit *B* to Bob for the teleportation attack. Alice will perform the BM on qubit *A*_1_ and *A*, which leads the entire system to be Eq. 15. As a result, we can determine the probabilities of the four qubit states in Eq. 15 after the BM by the IY basis and XZ basis are shown in Eqs 16 and 17, where Eqs 16 and 17 are the probabilities of states $\frac{1}{\sqrt{2}}{(|00\rangle +\frac{1}{\sqrt{2}}|10\rangle +\frac{1}{\sqrt{2}}|11\rangle )}_{{B}_{1}B}$ and $\frac{1}{\sqrt{2}}{(|01\rangle -\frac{1}{\sqrt{2}}|10\rangle +\frac{1}{\sqrt{2}}|11\rangle )}_{{B}_{1}B}$ in the IY basis, and Eqs 18 and 19 are the probabilities of states $\frac{1}{\sqrt{2}}{(|01\rangle +\frac{1}{\sqrt{2}}|10\rangle +\frac{1}{\sqrt{2}}|11\rangle )}_{{B}_{1}B}$ and $\frac{1}{\sqrt{2}}{(|00\rangle +\frac{1}{\sqrt{2}}|10\rangle -\frac{1}{\sqrt{2}}|11\rangle )}_{{B}_{1}B}$ in the XZ basis, where the bold numbers are the probabilities of dishonest Alice evading detection. After the above discussion of the defense strategy, we can determine the probability of Alice’s average escape detection of each entangled qubits pair is Eq. 20, and security level *ξ*_3_ is as given in Eq. 21, where *F* is the number of detected entangled qubit pairs. Therefore, Alice and Bob can decide security level *ξ*_3_, and whether they will continue the protocol according to the detection result. In summary, if the dishonest Alice only has the ability to perform single-qubit operations, then follow this protocol Bob can always have a probability to detect dishonesty one. Moreover, if the dishonest Alice can prepare Bell states or perform teleportation attacks, Alice’s cheating becomes more and more difficult because she has to have the technology to store the qubits received from Bob. However, such a long-term quantum storage technology is still a technical challenge and an open issue today. Even though when the long-term quantum storage technology can be built, the protocol still can intentionally delay the operation time between step 4 and step 7 to prevent these attacks.

15 $$(\begin{array}{c}\frac{1}{\sqrt{2}}{(|00\rangle +|+1\rangle )}_{{A}_{1}{B}_{1}}\\ \otimes \frac{1}{\sqrt{2}}{(|00\rangle +|11\rangle )}_{AB}\end{array})\mathop{\Rightarrow }\limits^{B{M}_{{A}_{1}A}}{(\begin{array}{c}|00\rangle (|00\rangle +\frac{1}{\sqrt{2}}|10\rangle +\frac{1}{\sqrt{2}}|11\rangle )\\ +|11\rangle (|01\rangle -\frac{1}{\sqrt{2}}|10\rangle +\frac{1}{\sqrt{2}}|11\rangle )\\ +|01\rangle (|01\rangle +\frac{1}{\sqrt{2}}|10\rangle +\frac{1}{\sqrt{2}}|11\rangle )\\ +|10\rangle (|00\rangle +\frac{1}{\sqrt{2}}|10\rangle -\frac{1}{\sqrt{2}}|11\rangle )\end{array})}_{{A}_{1}A{B}_{1}B}$$

16 $$(\begin{array}{cccc}\frac{1}{\sqrt{2}} & \frac{1}{2} & 0 & \frac{1}{2}\\ 0 & \frac{1}{2} & \frac{-1}{\sqrt{2}} & \frac{-1}{2}\\ \frac{1}{2} & 0 & \frac{1}{2} & \frac{-1}{\sqrt{2}}\\ \frac{-1}{2} & \frac{1}{\sqrt{2}} & \frac{1}{2} & 0\end{array})(\begin{array}{c}\frac{1}{\sqrt{2}}\\ 0\\ \frac{1}{2}\\ \frac{1}{2}\end{array})=(\begin{array}{c}0.750\\ -0.604\\ 0.250\\ -0.104\end{array})\mathop{\Rightarrow }\limits^{(\begin{array}{c}{|0.750|}^{2}\\ {|-0.604|}^{2}\\ {|0.250|}^{2}\\ {|-0.104|}^{2}\end{array})}(\begin{array}{c}{\bf{0.563}}\\ 0.364\\ 0.063\\ 0.011\end{array})$$

17 $$(\begin{array}{cccc}\frac{1}{\sqrt{2}} & \frac{1}{2} & 0 & \frac{1}{2}\\ 0 & \frac{1}{2} & \frac{-\,1}{\sqrt{2}} & \frac{-\,1}{2}\\ \frac{1}{2} & 0 & \frac{1}{2} & \frac{-\,1}{\sqrt{2}}\\ \frac{-\,1}{2} & \frac{1}{\sqrt{2}} & \frac{1}{2} & 0\end{array})(\begin{array}{c}0\\ \frac{1}{\sqrt{2}}\\ \frac{-\,1}{2}\\ \frac{1}{2}\end{array})=(\begin{array}{c}0.604\\ 0.457\\ -0.604\\ 0.250\end{array})\mathop{\Rightarrow }\limits^{(\begin{array}{c}{|0.604|}^{2}\\ {|0.457|}^{2}\\ {|-0.604|}^{2}\\ {|0.250|}^{2}\end{array})}(\begin{array}{c}0.364\\ {\bf{0.209}}\\ 0.364\\ 0.063\end{array})$$

18 $$(\begin{array}{cccc}0 & \frac{1}{2} & \frac{1}{\sqrt{2}} & \frac{1}{2}\\ \frac{1}{\sqrt{2}} & \frac{1}{2} & 0 & \frac{-\,1}{2}\\ \frac{1}{2} & \frac{-\,1}{\sqrt{2}} & \frac{1}{2} & 0\\ \frac{1}{2} & 0 & \frac{-1}{2} & \frac{1}{\sqrt{2}}\end{array})(\begin{array}{c}0\\ \frac{1}{\sqrt{2}}\\ \frac{1}{2}\\ \frac{1}{2}\end{array})=(\begin{array}{c}0.975\\ 0.104\\ -0.250\\ 0.104\end{array})\mathop{\Rightarrow }\limits^{(\begin{array}{c}{|0.975|}^{2}\\ {|0.104|}^{2}\\ {|-0.250|}^{2}\\ {|0.104|}^{2}\end{array})}(\begin{array}{c}{\bf{0.916}}\\ 0.011\\ 0.063\\ 0.011\end{array})$$

19 $$(\begin{array}{cccc}0 & \frac{1}{2} & \frac{1}{\sqrt{2}} & \frac{1}{2}\\ \frac{1}{\sqrt{2}} & \frac{1}{2} & 0 & \frac{-\,1}{2}\\ \frac{1}{2} & \frac{-\,1}{\sqrt{2}} & \frac{1}{2} & 0\\ \frac{1}{2} & 0 & \frac{-\,1}{2} & \frac{1}{\sqrt{2}}\end{array})(\begin{array}{c}\frac{1}{\sqrt{2}}\\ 0\\ \frac{1}{2}\\ \frac{-1}{2}\end{array})=(\begin{array}{c}0.104\\ 0.750\\ 0.604\\ -0.250\end{array})\mathop{\Rightarrow }\limits^{(\begin{array}{c}{|0.104|}^{2}\\ {|0.750|}^{2}\\ {|0.604|}^{2}\\ {|-0.250|}^{2}\end{array})}(\begin{array}{c}0.011\\ {\bf{0.563}}\\ 0.364\\ 0.063\end{array})$$

20 $$\frac{(\begin{array}{c}\mathrm{\ \ \ 0.563}+0.209\\ +0.916+0.563\end{array})}{4}=0.56275$$

21 $${\xi }_{3}=1-{\mathrm{(0.56275)}}^{F}$$

#### Bob’s cheating strategy

A dishonest Bob can prepare entangled qubits of the form |Φ^+^〉, as given in Eq. 1. In this way, he can learn the contents of all messages from Alice, with the results shown in Eq. 22, where the subscripts represent the qubit order; i.e., he can perfectly identify which operation Alice performed on qubit 1. However, only two states, |0〉 and |+〉, can be measured in the proposed protocol. Alice randomly selects *K* positions and asks Bob to publish the bases he prepared in step 2. If different measurement results are given, i.e., ∉ {|0〉, |+〉}, Bob is dishonest. The detection rate by legal agents, or the security level *ξ*_4_, is as given in Eq. 23.

22 $$\begin{array}{rcl}{I}_{1}{|{{\rm{\Phi }}}^{+}\rangle }_{12} & = & {I}_{1}\frac{1}{\sqrt{2}}{(|00\rangle +|11\rangle )}_{12}=\frac{1}{\sqrt{2}}{(|00\rangle +|11\rangle )}_{12}=\frac{1}{\sqrt{2}}{(|++\rangle +|--\rangle )}_{12}={|{{\rm{\Phi }}}^{+}\rangle }_{12}\\ {X}_{1}{|{{\rm{\Phi }}}^{+}\rangle }_{12} & = & {X}_{1}\frac{1}{\sqrt{2}}{(|00\rangle +|11\rangle )}_{12}=\frac{1}{\sqrt{2}}{(|01\rangle +|10\rangle )}_{12}=\frac{1}{\sqrt{2}}{(|++\rangle -|--\rangle )}_{12}={|{{\rm{\Psi }}}^{+}\rangle }_{12}\\ {Y}_{1}{|{{\rm{\Phi }}}^{+}\rangle }_{12} & = & {Y}_{1}\frac{1}{\sqrt{2}}{(|00\rangle +|11\rangle )}_{12}=\frac{1}{\sqrt{2}}{(|01\rangle -|10\rangle )}_{12}=\frac{1}{\sqrt{2}}{(|+-\rangle -|-+\rangle )}_{12}={|{{\rm{\Psi }}}^{+}\rangle }_{12}\\ {Z}_{1}{|{{\rm{\Phi }}}^{+}\rangle }_{12} & = & {Z}_{1}\frac{1}{\sqrt{2}}{(|00\rangle +|11\rangle )}_{12}=\frac{1}{\sqrt{2}}{(|00\rangle -|11\rangle )}_{12}=\frac{1}{\sqrt{2}}{(|+-\rangle +|-+\rangle )}_{12}={|{{\rm{\Phi }}}^{-}\rangle }_{12}\end{array}$$

23 $${\xi }_{4}=1-{(\frac{1}{2})}^{K}$$

### Efficiency Analysis

This section presents a performance comparison of the proposed protocol with three modern two-level-structured one-out-of-two QOT protocols^21,24,25^ based on Crépeau’s reduction^3^. The protocols of Wei Yang *et al*.^20^ and Yu-Guang Yang *et al*.^23,27^ are not considered because that of Wei Yang *et al*. may not work, whereas the first protocol of Yu-Guang Yang *et al*.^23^ involves an untrusted third party, and the second^27^ is a one-out-of-n QOT protocol for which the resource consumption for one-out-of-two QOT is similar to that of Li Yang’s protocol^21^. The three protocols considered for comparison^21,24,25^ have two-level structures in which one-out-of-two OT is built on all-or-nothing QOT. However, the probability *p* of all-or-nothing QOT (where *p* is the probability of the unambiguous key) is not always 50%. Significant quantum resources are required to build two classical keys (one unambiguous and the other unknown) using all-or-nothing QOT for one-out-of-two OT. In addition, every transmission should include decoy qubits for channel checking. Some protocols may need many transmissions, and many decoy qubits, to complete all-or-nothing QOT. For fairness, the security level *ξ*_1_ is ensured to be at least 99.9999% by using 50 decoy qubits for each transmission. Then, the most important indicators are the conversion efficiency between two OT protocols and the number of transmissions (which indirectly affects the number of decoy qubits). The total cost of each protocol, as calculated under the requirement that *R* message bits are received, is given in Table 3. Here, only the quantum cost without loyalty testing is considered; for fairness, we do not include the cost for loyalty testing because two of the other one-out-of-two QOT protocols^21,24^ do not consider any loyalty testing, which means that the sender and receiver may not truly trust each other, whereas the loyalty testing of the third protocol^24^ requires the consumption of a large number of qubits, making the quantum cost difficult to calculate. Detailed descriptions of the protocols considered for comparison^21,24,25^ are given below.

**Table 3:** Performance comparison of three modern 1–2 QOT protocols^21,24,25^ with the proposed protocol.

1–2 QOT Protocols	^a^Quantum Resources for One Message Bit	^b^Number of Transmissions	^c^Decoy Qubits	^d^Total Cost
Yang’s protocol^21^	4	1	50	4 × *R* + 50
YYLSZ protocol^24^	4	2	2 × 50	4 × *R* + 2 × 50
YSW protocol^25^	4	1	50	4 × *R* + 50
Our protocol	1	2	2 × 50	*R* + 2 × 50

^a^Quantum Resources for One Message Bit: Number of quantum resources consumed for each received bit, without decoy qubits.

^b^Number of Transmissions: Number of transmissions for one sequence.

^c^Decoy Qubits: Number of decoy qubits, considering the number of transmissions.

^d^Total Cost: The total average quantum resource consumption for *R* received bits.

#### Yang’s protocol

This protocol^21^ uses the B92^22^ protocol as the all-or-nothing QOT protocol on which it is based. Therefore, it requires four qubits on average to obtain an unambiguous key and only one transmission. However, the cited study focused more on the bit-commitment protocol than on the OT protocol, with no further security analysis of the QOT protocol or strategies for detecting eavesdroppers. Therefore, no strategy was provided for loyalty testing between Alice and Bob. In addition, the number of decoy qubits is computed as described above because the original detection strategy of B92^22^ is less efficient. Therefore, the same detection strategy with decoy qubits is used. As a result, the total quantum cost is 4 × *R* + 50.

#### YYLSZ protocol

This protocol^24^ also requires at least four qubits on average to obtain an unambiguous key using its all-or-nothing QOT strategy, i.e., $p=\frac{1}{4}$. In the all-or-nothing QOT protocol, Alice first sends a sequence to Bob, and Bob then sends it back after his measurement, which requires two transmissions. After this, Alice can test Bob’s loyalty by observing the probability of occurrence of states |+〉 and |−〉. Note that this strategy is based on the law of large number and will consume a large number of qubits. By contrast, Bob cannot really test Alice’s loyalty; he is only able to detect errors in the later application of the one-out-of-two OT protocol. The overall cost of the protocol is 4 × *R* + 2 × 50.

#### YSW protocol

This protocol^25^ reduces BB84^6^ to B92^22^. It uses the BB84^6^ strategy and the publication of additional state information to allow Bob to generate unambiguous keys as in B92^22^. It also requires four qubits for the generation of an unambiguous key. In addition, it requires one transmission to complete all-or-nothing QOT. However, it does not include a loyalty testing method for the all-or-nothing QOT stage. Errors may be detected at the application level. The overall cost of the protocol is 4 × *R* + 50.

#### Proposed protocol

The proposed protocol is based directly on quantum resources and consumes one qubit for each received bit. The proposed protocol is more efficient than the others^21,24,25^, with a probability *p* of $\frac{1}{4}$. The proposed protocol requires two transmissions between Alice and Bob. The overall cost of the proposed protocol is *R* + 2 × 50.

**Figure 1:**
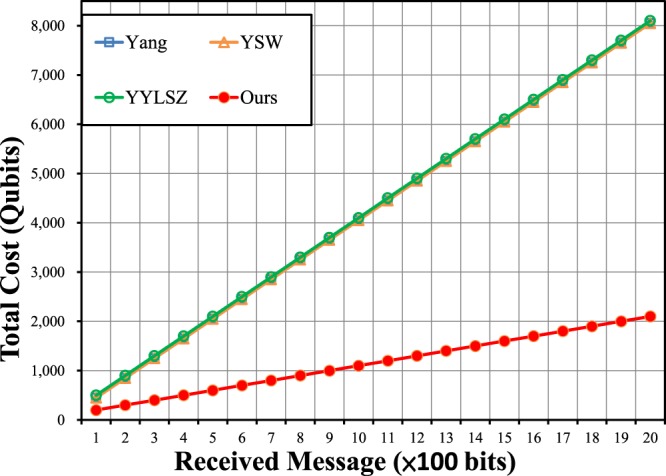
Illustration of the comparison results.

As seen from Table 3, the proposed protocol is the most efficient among all of the compared protocols^21,24,25^; this is also shown in Fig. 1. This result demonstrates that building a one-out-of-two QOT protocol directly is more efficient than Crépeau’s reduction^3^, which requires a two-level structure.

## Discussion

In conclusion, there three important contributions of the proposed method. First, to the best of our knowledge, the proposed protocol is the first one-out-of-two QOT protocol to be designed directly based on quantum properties without relying on all-or-nothing QOT, and it has been proven to be secure and not subject to Lo’s no-go theorem^15^. A simple and efficient one-out-of-two QOT protocol with single nonorthogonal qubits, in which one of the two messages is discarded automatically, has been successfully developed based on the most basic properties of quantum machines. Second, the proposed protocol can effectively prevent both external and internal attacks, as proven by a detailed security analysis. Regarding internal attacks, an important feature of the proposed protocol is that loyalty testing is applied to provide security against internal attacks by a dishonest Alice or Bob; the dishonest one can always be detected at the loyalty testing stages with safety parameters *ξ*_2_, *ξ*_3_ and *ξ*_4_, respectively. Finally, the proposed protocol has a lower cost and is more efficient than many traditional protocols based on a two-level structure. In addition, as this protocol uses only a single qubit, it is easily implemented.

## Data Availability

No datasets were generated or analysed during the current study.
