# Author Correction: One-out-of-two Quantum Oblivious Transfer based on Nonorthogonal States

**DOI:** 10.1038/s41598-018-37934-4

**Published:** 2019-03-28

**Authors:** Yao-Hsin Chou, Guo-Jyun Zeng, Shu-Yu Kuo

**Affiliations:** Department of Computer Science and Information Engineering, National Chi Nan University, Puli, 54561 Taiwan

Correction to: *Scientific Reports* 10.1038/s41598-018-32838-9, published online 29 October 2018

The Acknowledgements section in this Article is incomplete.

“This research was partially supported by the Ministry of Science and Technology (MOST), Taiwan, R.O.C., under Grant no. MOST 104-2221-E-260-011 & 105-2221-E-260-021.”

should read:

“This research was supported in part by the Ministry of Science and Technology of R.O.C. under Grants MOST 106-2221-E-260-019, 107-2221-E-260-019- MY2, 107-2918-I-260-001, and MOST 107-2917-I-564-031 and in part by the National Taiwan University under Grant 107L892903.”

